# Patient Adherence for Oral Combination Therapies in Diabetes Management: A Scoping Review

**DOI:** 10.1002/hsr2.70780

**Published:** 2025-04-29

**Authors:** Maryam Zarrinkamar, Mojgan Geran, Mohammad Geran

**Affiliations:** ^1^ Department of Family Medicine, Diabetes Research Center Mazandaran University of Medical Sciences Sari Iran; ^2^ Health Department Mazandaran University of Medical Sciences Sari Iran

**Keywords:** combination therapy, fixed‐dose combination, medication adherence, oral antihyperglycemic agents, Type 2 Diabetes Mellitus

## Abstract

**Background and Aims:**

Diabetes imposes a global healthcare burden, with Type 2 Diabetes Mellitus (T2DM) escalating due to demographic shifts and lifestyle changes. Combination therapies offer promise in managing T2DM, yet patient adherence remains a challenge. This scoping review aims to explore patient adherence to combination therapies in T2DM management.

**Materials and Methods:**

This scoping review followed the Preferred Reporting Items for Systematic reviews and Meta‐Analyses extension for Scoping Reviews guidelines. Electronic databases including PubMed, Scopus, and Web of Science were searched until July 10, 2024. Studies focused on patients with T2DM prescribed oral combination therapies were included. Data extraction and synthesis were conducted to identify adherence patterns, influencers, and strategies.

**Results:**

The review identified nine eligible studies spanning from 2004 to 2021, primarily retrospective cohort and cross‐sectional designs. Adherence assessment methods varied, with medication possession ratio being the most common. Both dual (loose‐dose) and fixed‐dose combination therapies were explored. Adherence rates varied across studies and therapies, influenced by factors such as glycemic control, weight management, economic considerations, complexity of regimens, and demographic factors. Bibliometric analysis revealed diverse geographic origins of the included studies and varied adherence assessment methods. Dual therapy regimens demonstrated adherence rates ranging from 49% to 80.8%, while FDC therapies showed adherence rates ranging from 60.3% to 98.9%. Factors influencing adherence included glycemic control, weight management, economic considerations, complexity of regimens, and demographic factors.

**Conclusion:**

Patient adherence to oral combination therapies in diabetes management is complex, affected by clinical, economic, and psychosocial factors. Addressing these issues is crucial for enhancing treatment outcomes and alleviating the burden of diabetes. A patient‐centered approach and innovative strategies can empower patients to adhere to medication regimens, improving health outcomes and quality of life.

## 1 Introduction

Diabetes, a chronic metabolic disorder characterized by elevated blood glucose levels, continues to impose a substantial burden on global healthcare systems and individual well‐being [1]. In contemporary times, Type 2 Diabetes Mellitus (T2DM) has evolved into a health condition with significant economic and societal ramifications [2]. The global prevalence and frequency of this ailment are on a swift upward trajectory, attributable to factors such as demographic shifts towards an aging population, alterations in dietary patterns, and a progressively sedentary way of life [3, 4]. As per the findings of the Global Burden of Diseases, Injuries, and Risk Factors Study (GBD) 2023, diabetes ranked as the eighth primary contributor to mortality and incapacitation collectively worldwide. In 2023, close to 529 million individuals spanning various countries and age brackets were reported to be afflicted by this condition [5]. Diabetes often incurs substantial costs due to its propensity for complications over time, necessitating increased resource allocation and significantly impacting healthcare system [6, 7]. As time progresses and glycemic control declines, patients who are not effectively managed with monotherapy often necessitate treatment intensification through the incorporation of multiple oral antihyperglycemic agents [8]. In the midst of the diverse array of management strategies, the adoption of combination therapies has emerged as a crucial tactic for attaining optimal glycemic control and reducing associated complications [9]. In the process of selecting therapy, patient adherence plays a pivotal role, especially when faced with multiple suitable alternative medications for a medical condition [10]. When expressing adherence, patients must assess the anticipated therapeutic benefits against potential side effects [10]. However, since both the therapeutic response and side effects can vary significantly among individuals, it becomes challenging for patients and their physicians to determine the optimal treatment for them [9]. Studies have indicated that adherence to antihyperglycemic medications is commonly inadequate, frequently failing to exceed the typical threshold of 80% [11]. One potential explanation for suboptimal adherence may lie in the complexity of medication regimens and the burden of pill intake associated with the management of T2DM therapy [12]. Initially, therapy often begins with monotherapy but may progress to dual or triple therapy, each medication leveraging distinct mechanisms of action to achieve additive or synergistic effects [13]. Simplifying pharmacological treatment for patients requiring multiple medications can be achieved through the utilization of fixed‐dose combinations (FDCs). These combinations integrate two or more active components into a single dosage form, thereby diminishing pill burden and potentially enhancing adherence to treatment [13]. However, FDCs may not accommodate individual patient needs, as they combine specific doses of multiple drugs into a single formulation. This lack of flexibility can be a barrier, especially when patients require adjustments to their medication regimen [14]. In this scoping examination, we undertake a thorough investigation into patient adherence concerning combination therapies used in managing diabetes. By amalgamating current literature, we probe into the complexities of adherence patterns, recognizing obstacles, enablers, and strategies influencing patient adherence. Acknowledging the pivotal role of adherence in achieving effective diabetes management, this review endeavors to offer perspectives on enhancing treatment effectiveness and promoting patient‐centric care approaches.

## 2 Materials and Methods

### 2.1 Reporting

This scoping review followed the Preferred Reporting Items for Systematic reviews and Meta‐Analyses extension for Scoping Reviews (PRISMA‐ScR) guidelines to ensure transparency and completeness in reporting the methods and findings [15].

### 2.2 Search Strategy

A thorough exploration of the existing literature was carried out through electronic databases such as PubMed, Scopus, and Web of Science, covering studies from their inception until July 10th, 2024. The search strategy employed a combination of keywords, Medical Subject Headings (MeSH) terms, and relevant synonyms, as outlined in Table 1.

**Table 1:** Searching strategy for different databases.

Electronic database		
Pubmed search query	Scopus search query	Web of science search query
((Type 2 Diabetes [title/abstract]) OR (Noninsulin‐Dependent Diabetes Mellitus [title/abstract]) OR (Type 2 Diabetes Mellitus [title/abstract])) AND ((patient preference [title/abstract]) OR (patient choice [title/abstract]) OR (patient decision making [title/abstract]) OR (patient adherence [title/abstract]) OR (Client Adherence [title/abstract]) OR (Client Compliance [title/abstract]) OR (Non‐Adherent Patient [title/abstract]) OR (Patient Cooperation [title/abstract]) OR (Patient Non‐Adherence [title/abstract]) OR (Patient Noncompliance [title/abstract]) OR (Patient Nonadherence [title/abstract]) OR (Patient Noncompliance [title/abstract]) OR (Therapeutic Compliance [title/abstract]) OR (Treatment Compliance [title/abstract]) OR (adherence [title/abstract]) OR (compliance [title/abstract]) OR (patient persistence [title/abstract]) OR (persistence [title/abstract])) AND ((metformin/sitagliptin [title/abstract]) OR (empagliflozin/metformin [title/abstract]) OR (empagliflozin/linagliptin [title/abstract]) OR (dapagliflozin/metformin [title/abstract]) OR (metformin/saxagliptin [title/abstract]) OR (linagliptin/metformin [title/abstract]) OR (metformin/pioglitazone [title/abstract]) OR (metformin/rosiglitazone [title/abstract]) OR (alogliptin/pioglitazone [title/abstract]) OR (canagliflozin/metformin [title/abstract]) OR (glyburide/metformin [title/abstract]) OR (ertugliflozin/sitagliptin [title/abstract]) OR (metformin/repaglinide [title/abstract]) OR (glipizide/metformin [title/abstract]) OR (glimepiride/pioglitazone [title/abstract]) OR (glimepiride/rosiglitazone [title/abstract]) OR (metformin + sitagliptin [title/abstract]) OR (empagliflozin + metformin [title/abstract]) OR (empagliflozin + linagliptin [title/abstract]) OR (dapagliflozin + metformin [title/abstract]) OR (metformin + saxagliptin [title/abstract]) OR (linagliptin + metformin [title/abstract]) OR (metformin + pioglitazone [title/abstract]) OR (metformin + rosiglitazone [title/abstract]) OR (alogliptin + pioglitazone [title/abstract]) OR (canagliflozin + metformin [title/abstract]) OR (glyburide + metformin [title/abstract]) OR (ertugliflozin + sitagliptin [title/abstract]) OR (metformin + repaglinide [title/abstract]) OR (glipizide + metformin [title/abstract]) OR (glimepiride + pioglitazone [title/abstract]) OR (glimepiride + rosiglitazone [title/abstract]) OR (metformin vs. sitagliptin [title/abstract]) OR (empagliflozin vs. metformin [title/abstract]) OR (empagliflozin vs. linagliptin [title/abstract]) OR (dapagliflozin vs. metformin [title/abstract]) OR (metformin vs. saxagliptin [title/abstract]) OR (linagliptin vs. metformin [title/abstract]) OR (metformin vs. pioglitazone [title/abstract]) OR (metformin vs. rosiglitazone [title/abstract]) OR (alogliptin vs. pioglitazone [title/abstract]) OR (canagliflozin vs. metformin [title/abstract]) OR (glyburide vs. metformin [title/abstract]) OR (ertugliflozin vs. sitagliptin [title/abstract]) OR (metformin vs. repaglinide [title/abstract]) OR (glipizide vs. metformin [title/abstract]) OR (glimepiride vs. pioglitazone [title/abstract]) OR (glimepiride vs. rosiglitazone [title/abstract]) OR (combination regimen [title/abstract]) OR (combination therapy [title/abstract]) OR (dual therapy [title/abstract]) OR (fixed‐dose combination [title/abstract]))	(TITLE‐ABS‐KEY((“Type 2 Diabetes” OR “Noninsulin‐Dependent Diabetes Mellitus” OR “Type 2 Diabetes Mellitus”) AND (“patient preference” OR “patient choice” OR “patient decision making” OR “patient adherence” OR “Client Adherence” OR “Client Compliance” OR “Non‐Adherent Patient” OR “Patient Cooperation” OR “Patient Non‐Adherence“ OR “Patient Noncompliance” OR “Patient Nonadherence” OR “Patient Noncompliance” OR “Therapeutic Compliance” OR “Treatment Compliance” OR “adherence” OR “compliance” OR “patient persistence” OR “persistence”) AND (“metformin/sitagliptin” OR “empagliflozin/metformin” OR “empagliflozin/linagliptin” OR “dapagliflozin/metformin” OR “metformin/saxagliptin” OR “linagliptin/metformin” OR “metformin/pioglitazone” OR “metformin/rosiglitazone” OR “alogliptin/pioglitazone” OR “canagliflozin/metformin” OR “glyburide/metformin” OR “ertugliflozin/sitagliptin” OR “metformin/repaglinide” OR “glipizide/metformin” OR “glimepiride/pioglitazone” OR “glimepiride/rosiglitazone” OR “metformin + sitagliptin” OR “empagliflozin + metformin” OR “empagliflozin + linagliptin” OR “dapagliflozin + metformin” OR “metformin + saxagliptin” OR “linagliptin + metformin” OR “metformin + pioglitazone” OR “metformin + rosiglitazone” OR “alogliptin + pioglitazone” OR “canagliflozin + metformin” OR “glyburide + metformin” OR “ertugliflozin + sitagliptin” OR “metformin + repaglinide” OR “glipizide + metformin” OR “glimepiride + pioglitazone” OR “glimepiride + rosiglitazone” OR “metformin vs. sitagliptin” OR “empagliflozin vs. metformin” OR “empagliflozin vs. linagliptin” OR “dapagliflozin vs. metformin” OR “metformin vs. saxagliptin” OR “linagliptin vs. metformin” OR “metformin vs. pioglitazone” OR “metformin vs. rosiglitazone” OR “alogliptin vs. pioglitazone” OR “canagliflozin vs. metformin” OR “glyburide vs. metformin” OR “ertugliflozin vs. sitagliptin” OR “metformin vs. repaglinide” OR “glipizide vs. metformin” OR “glimepiride vs. pioglitazone” OR “glimepiride vs. rosiglitazone” OR “combination regimen” OR “combination therapy” OR “dual therapy” OR “fixed‐dose combination”)))	(TS=(“Type 2 Diabetes” OR “Noninsulin‐Dependent Diabetes Mellitus” OR “Type 2 Diabetes Mellitus”)) AND (TS=(“patient preference” OR “patient choice” OR “patient decision making” OR “patient adherence” OR “Client Adherence” OR “Client Compliance” OR “Non‐Adherent Patient” OR “Patient Cooperation” OR “Patient Non‐Adherence“ OR “Patient Noncompliance” OR “Patient Nonadherence” OR “Patient Noncompliance” OR “Therapeutic Compliance” OR “Treatment Compliance” OR “adherence” OR “compliance” OR “patient persistence” OR “persistence”)) AND (TS=(“metformin/sitagliptin” OR “empagliflozin/metformin” OR “empagliflozin/linagliptin” OR “dapagliflozin/metformin” OR “metformin/saxagliptin” OR “linagliptin/metformin” OR “metformin/pioglitazone” OR “metformin/rosiglitazone” OR “alogliptin/pioglitazone” OR “canagliflozin/metformin” OR “glyburide/metformin” OR “ertugliflozin/sitagliptin” OR “metformin/repaglinide” OR “glipizide/metformin” OR “glimepiride/pioglitazone” OR “glimepiride/rosiglitazone” OR “metformin + sitagliptin” OR “empagliflozin + metformin” OR “empagliflozin + linagliptin” OR “dapagliflozin + metformin” OR “metformin + saxagliptin” OR “linagliptin + metformin” OR “metformin + pioglitazone” OR “metformin + rosiglitazone” OR “alogliptin + pioglitazone” OR “canagliflozin + metformin” OR “glyburide + metformin” OR “ertugliflozin + sitagliptin” OR “metformin + repaglinide” OR “glipizide + metformin” OR “glimepiride + pioglitazone” OR “glimepiride + rosiglitazone” OR “metformin vs. sitagliptin” OR “empagliflozin vs. metformin” OR “empagliflozin vs. linagliptin” OR “dapagliflozin vs. metformin” OR “metformin vs. saxagliptin” OR “linagliptin vs. metformin” OR “metformin vs. pioglitazone” OR “metformin vs. rosiglitazone” OR “alogliptin vs. pioglitazone” OR “canagliflozin vs. metformin” OR “glyburide vs. metformin” OR “ertugliflozin vs. sitagliptin” OR “metformin vs. repaglinide” OR “glipizide vs. metformin” OR “glimepiride vs. pioglitazone” OR “glimepiride vs. rosiglitazone” OR “combination regimen” OR “combination therapy” OR “dual therapy” OR “fixed‐dose combination”))

### 2.3 Inclusion and Exclusion Criteria

#### 2.3.1 Patient

The focus of this review is on patients diagnosed with T2DM. These patients are prescribed oral combination therapies, which involve the simultaneous use of multiple medications to control blood sugar levels and manage the disease. Understanding patient adherence is essential as it directly influences treatment efficacy and overall health outcomes in individuals with diabetes.

#### 2.3.2 Concept

The central concept of this review is to examine the extent to which patients with T2DM adhere to oral combination therapies prescribed for disease management. Adherence refers to the degree to which patients follow their prescribed treatment regimen, including medication intake, dosage, frequency, and timing. By exploring patient adherence in the context of oral combination therapies, the review aims to identify factors influencing adherence patterns and strategies to improve treatment compliance among individuals with diabetes.

#### 2.3.3 Context

The review takes place within the broader context of diabetes management, where achieving and maintaining optimal glycemic control is crucial to prevent complications and improve quality of life for patients. Oral combination therapies have become increasingly common in diabetes management due to their potential to simplify treatment regimens and enhance therapeutic outcomes [16]. However, the effectiveness of these therapies relies heavily on patient adherence. By conducting a scoping review, the study seeks to provide a comprehensive overview of existing literature on patient adherence to oral combination therapies in diabetes management, thus informing future research directions and clinical practice guidelines in this field. Both quantitative and qualitative study designs were eligible for inclusion. Studies that did not report outcomes related to adherence or concentrated solely on monotherapy or combination therapy with injectable medications (insulin therapy) were excluded from the review. Also, only English papers were included in the manuscript and non‐English papers were excluded.

### 2.4 Study Selection

Two independent reviewers assessed the relevance of titles and abstracts to the research objectives, engaging in a thorough examination to ensure no pertinent studies were overlooked. This initial screening phase, characterized by careful scrutiny, served as a crucial filter in identifying potentially suitable research contributions. Subsequent to this rigorous evaluation, the full texts of studies identified as potentially eligible underwent a comprehensive retrieval process for further scrutiny and analysis. This subsequent stage involved a review of each full text to gauge its alignment with the research aims and adherence to the predetermined inclusion criteria. This detailed evaluation process, marked by attention to detail, aimed to uphold the integrity and robustness of the study's selection process, thus enhancing the reliability and validity of the findings.

### 2.5 Data Extraction

Data extraction from the included studies was conducted utilizing a standardized form to ensure consistency. The extracted information encompassed various aspects such as study design, the country where the study was conducted, participant demographics, intervention specifics including types of combination therapies employed, methods utilized for measuring adherence, adherence rates observed, factors that influenced adherence, and outcomes pertaining to the management of diabetes.

### 2.6 Data Synthesis

The findings extracted from the included studies were gathered through a narrative synthesis approach. This involved a comprehensive examination of the data to identify recurring patterns and overarching themes pertaining to patient adherence in the context of combination therapies for diabetes management. Through this synthesis process, key insights and perspectives regarding the complexities and determinants of adherence were discerned and succinctly summarized to provide a comprehensive overview of the literature on this subject.

## 3 Data Synthesis

We demonstrate the outcomes of the exploration and evaluation of literature, define the studies that fulfilled the criteria for inclusion, and outline the findings of these studies concerning the adherence of T2DM patients to combination antihyperglycemic medications. Additionally, we extended our analysis to encompass a descriptive numerical summary, shedding light on the varying levels of adherence observed among patients undergoing combination therapy for T2DM. This comprehensive approach offers a deeper understanding of the adherence patterns within this patient population, facilitating nuanced insights into treatment practices and potential avenues for improvement. In contrast to a systematic review, the aim of this review diverges from making clinical practice recommendations or assessing intervention effectiveness. Instead, its objective is to delineate and encapsulate the evidence panorama regarding a specific topic, marking its inaugural exploration. Consequently, a risk‐of‐bias evaluation isn't undertaken for the encompassed studies, aligning with the framework of scoping reviews and adhering to contemporary methodological guidelines advocated by Joanna Briggs Institute [17].

## 4 Results

### 4.1 Searching Strategy Results

**Figure 1:**
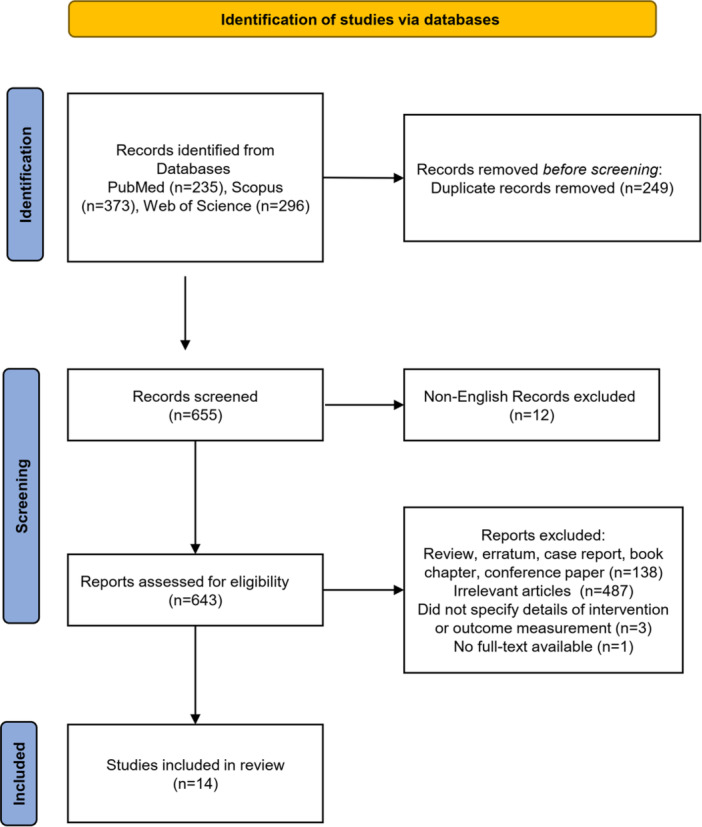
PRISMA flowchart.

In the execution of the scoping review, an initial search was conducted across three eminent databases: PubMed, Scopus, and Web of Science, yielding a cumulative total of 904 records. The distribution across these databases revealed a significant presence in Scopus (*n* = 373), followed by PubMed (*n* = 235), and Web of Science (*n* = 296). Before the commencement of eligibility screening, a curation process was undertaken, resulting in the identification and removal of 249 duplicate records. This strategic purging operation was deemed essential to streamline the dataset, effectively expunging redundancy and fortifying the integrity of the review process. Subsequent to the elimination of duplicates, the dataset was honed to a more manageable scale, comprising 665 unique records ripe for screening. Within screening, the application of stringent language criteria led to the exclusion of 12 non‐English records, aligning with the predetermined parameters of the review. With the dataset thus refined, the screening process was commenced, subjecting 643 records to rigorous scrutiny against predefined inclusion and exclusion criteria. The evaluative phase unearthed a multifaceted landscape, wherein 138 records were deemed ineligible due to their classification as review, erratum, case report, book chapter, and conference paper. Furthermore, an additional 487 articles were deemed tangential to the scope of the review, failing to align with the core objectives and parameters. A granular analysis during this phase unearthed nuances, wherein three records faced exclusion due to ambiguous intervention or outcome measurement specifications, underscoring the meticulousness applied in the eligibility assessment. also access to the full‐text of one paper was not possible. Emerging from this evaluation process were 14 studies that successfully navigated the stringent criteria for inclusion. This selects subset, distilled from the initial pool of records, now stands as the cornerstone for subsequent analysis and synthesis, heralding the commencement of a journey towards comprehensive insights and nuanced understandings within the realm under review (Figure 1).

### 4.2 Bibliometric Analysis

**Figure 2:**
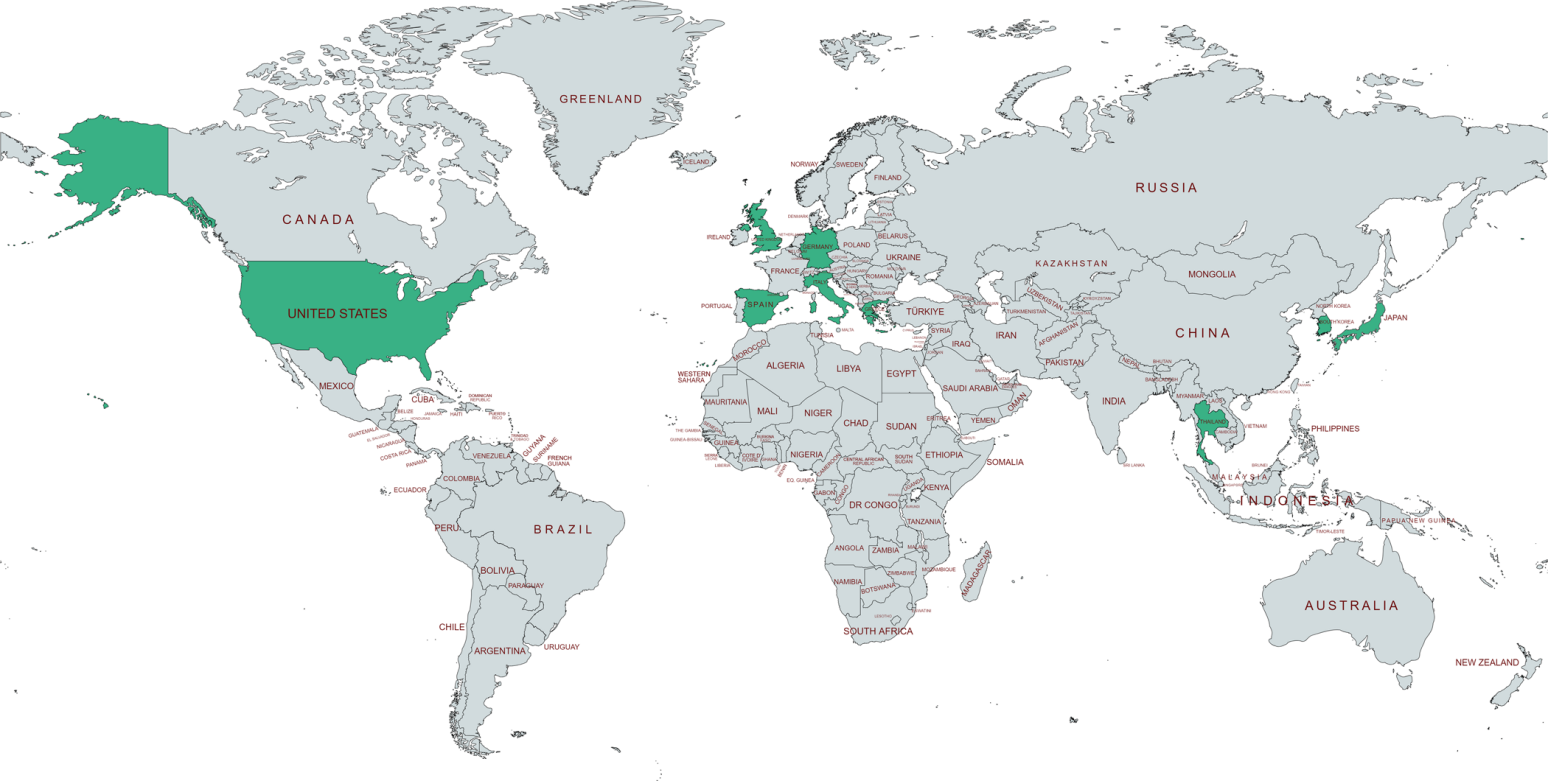
Locations of included studies measuring adherence of patients to combination antidiabetic drugs. Green countries are included in the study.

**Figure 3:**
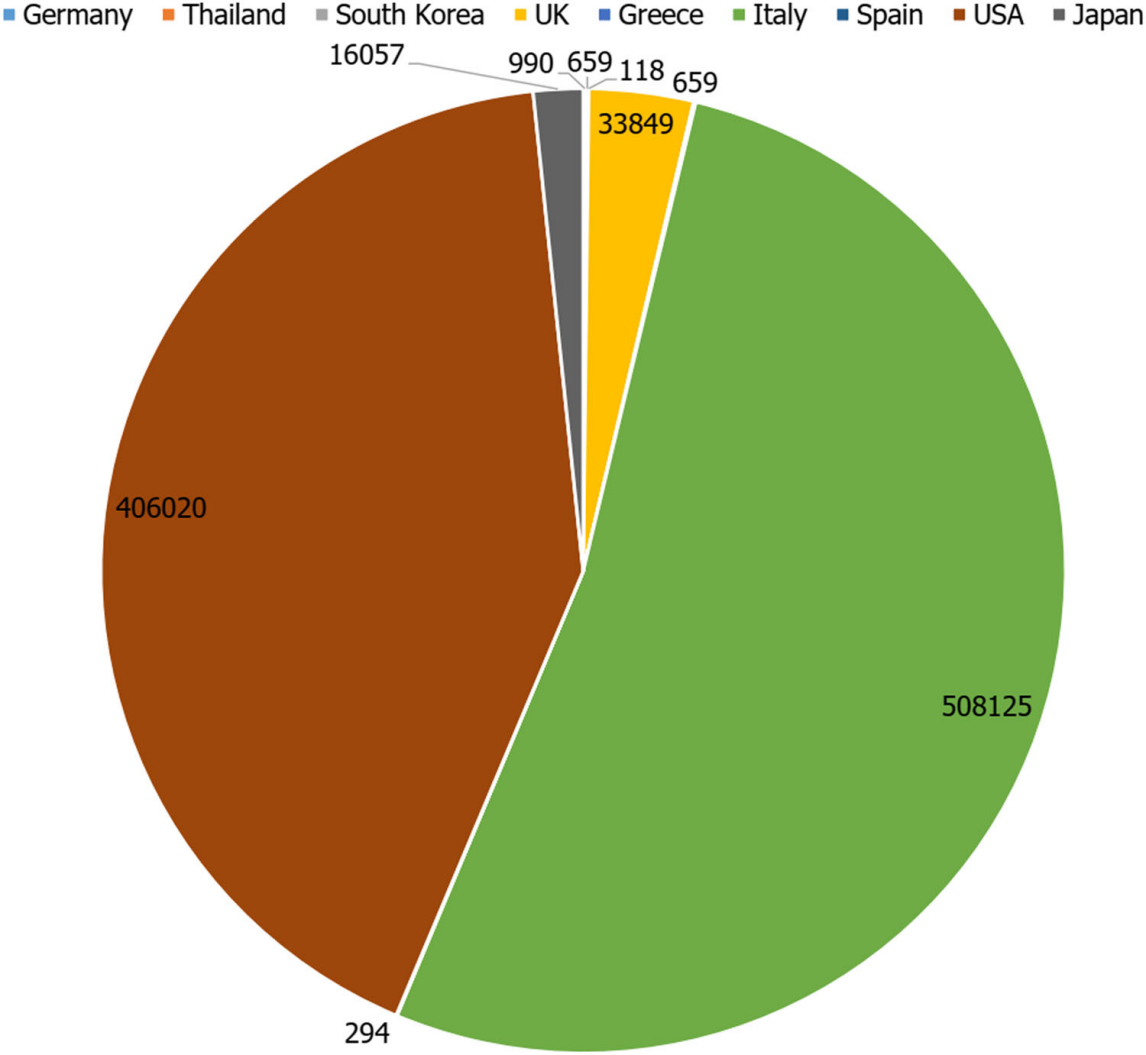
Population engaged in the study from different countries.

Table 2 showcased the attributes of the studies incorporated into our investigation. A total number of 576,150 people were studied in the included research. While the search strategy did not impose a temporal constraint and extended until July 10th 2024, the studies included in our analysis spanned from 2004 to 2021. These studies originated from various countries including Germany, Thailand, South Korea, the UK, Greece, Italy, Spain, Japan and the USA [18, 19, 20, 21, 22, 23, 24, 25, 26, 27, 28, 29, 30] (Figure 2). Most of the studies were retrospective cohort studies (*n* = 11) [21, 24, 25, 28, 29]. Two research were cross‐sectional studies [27, 30], and just one research was randomized controlled trial study [23]. The bibliometric data of the included research is demonstrated in Figure 3. The most common method for the assessment of adherence of T2DM patients to combination antihyperglycemic therapy was Medication Possession Ratio (MPR) [19, 20, 21, 22, 24, 28, 29]. Other adherence evaluation methods were proportion of days covered (PDC) [18, 25], Self‐Report Adherence and Barriers Questionnaire [27], Compliance questionnaire [26], Medication Event Monitoring System (MEMS) [23] and Morisky‐Green questionnaire [30].

**Table 2:** Data extraction of included studies.

Author/date	Country	Type of study	Patients	Intervention	Duration	Adherence measurement method	Adherence/compliance rate	Factors influencing adherence/compliance
Böhm, 2021	Germany	Retrospective cohort study	990 patients with T2DM	FDC vs. LDC (sitagliptin and Met)	2013–2017	PDC	Increased adherence observed in FDC group compared to LDC group over three years (year 1: 22%; year 2: 25%; year 3: 29%)	Pill burden, concomitant disease, baseline adherence
Satirapoj, 2020	Thailand	Cross‐sectional	659 patients with T2DM	SU monotherapy vs. SU and Met combination	2013–2015	Self‐Report Adherence and Barriers Questionnaire	52.2% of the patients achieved compliance; There was no significant difference between SU and SU+Met groups in compliance	Hypoglycaemia, weight gain, medication side effects
Nishimura, 2019	Japan	Retrospective, longitudinal cohort study	16057 patients with T2DM	**FDC vs. LDC:** TZD+ Biguanides, TZD+SU, TZD+DPP‐4i, DPP‐4i+ Biguanides, Alpha‐glucosidase+ Glinide	2011–2015	PDC	FDC (70.4) and LDC (66.2%) in Japan Medical Data Center and FDC (75.6) and LDC (55.7%) in Medical Data Vision	Cost of drug, Pill burden, Patient age
Kim, 2018	South Korea	Randomized Controlled Trial (NCT01620489)	118 patients with T2DM	GM‐SR once‐daily or GM‐IR BID	24 weeks	MEMS	GM‐SR: 91.7%, GM‐IR: 88.6%	Frequency of dosing
Gordon, 2018	UK	Retrospective cohort study	33849 patients with T2DM	OHA (mono, dual or triple) therapy **Monotherapy**: MET, SU, DPP‐4i; **Dual therapy**: Met+SU, Met+DPP‐4i, Met+TZD, MET+SGLT‐2i, SU+DPP‐4i, **Triple therapy**: MET+SU+DPP‐4i, MET+SU+TZD, MET+DPP‐4i+SGLT‐2i, Met+DPP‐4i+TZD, MET+SU+SGLT‐2i	2008–2016	MPR	Monotherapy (81.6%), dual‐therapy (80.8%), and triple‐therapy (80.8%)	Hypoglycemia rates, patient age, baseline HbA1c, change in BMI, change in total cholesterol
Rombopoulos, 2015	Greece	Retrospective cohort study	659 patients with T2DM	**FDC vs. LDC:** Vildagliptin/metformin	24 weeks	Compliance questionnaire	LDC (84.6%) and FDC (98.9%)	Remembering medication names, difficulty swallowing
Lokhandwala, 2015	USA	Retrospective observational cohort study	23361 patients with T2DM	**FDC vs. LDC:** DPP‐4i+ Biguanides DPP‐4i+ TZD, Meglitinides+ Biguanides, SU+TZD, SU+ Biguanides, TZD+ Biguanides	January 1, 2009 to December 31, 2013	MPR	FDC (67.9%) vs. LDC (73.4%)	Hypoglycemia, Cost of drug (LDC was lower)
Gaddi, 2014	Italy	Retrospective cohort study	169375 patients with T2DM	**Monotherapy**: Met, Gliclazide, Glimepiride, Repaglinide, Glibenclamide, Pioglitazone, Gliquidone, Glipizide, Rosiglitazone, Sitagliptin, Exenatide, Clorpropamide, Acarbose, Vildagliptin **FDC:** Met+SU, Fenformin+SU, Met+rosiglitazone, Met+pioglitazone **LDC:** Biguanides+SU Biguanides+TZD, SU+TZD	2008–2013	MPR	Monotherapy: 43.9% FDC: 68.5% LDC: 60.3%	Glycemic control, ease of use of FDC in a long time
Vittorino, 2014	Italy	Retrospective cohort study	169,375 patients with T2DM	**Monotherapy:** Met, Gliclazide, Glimepiride, Repaglinide, Glibenclamide, Pioglitazone, Gliquidone, Glipizide, Rosiglitazone, Sitagliptin, Exenatide, Clorpropamide, Acarbose, Vildagliptin **FDC:** Met+SU, Fenformin+SU, Met+Rosiglitazone, Met+Pioglitazone **LDC:** Biguanides+SU, Biguanides+TZD, SU+TZD	January 1 2008–December 31 2008	MPR	Monotherapy (68.5%), FDC (60.3%), LDC (43.9%)	Patient demographics and Pill burden
Colombo, 2012	Italy	Retrospective cohort study	169,375 patients with T2DM	**Monotherapy**: TZD, Sulfonamides, Met, Fenformin, DPP‐4i, Alpha glucosidase inhibitors **FDC:** Fenformin+sulfonamides, Met+sulfonamides, Met+rosiglitazone, Glimepiride+rosiglitazone, Met+pioglitazone, Glimepiride+pioglitazone, Met+sitagliptin, Met+vildagliptin	January 1, to December 31, 2008	MPR	Best adherence for monotherapy: glimepiride (70.5%) Best adherence for FDC: Met+ pioglitazone (75.5%)	Cost of drug (FDC is lower)
Thayer, 2010	USA	Retrospective cohort study	16490 patients with T2DM	**Monotherapy:** SU and rosiglitazone **FDC:** Rosiglitazone/Glimepiride **LDC:** SU and rosiglitazone	January 1, 2006, to September 30, 2006	MPR	Monotherapy/FDCT (−2%), Monotherapy/LDC (−1%), LDC/FDCT Mean MPR improved by 0.10	Pill burden, Patient age, glycemic control
Yurgin, 2008	Spain	Cross‐sectional study	294 patients with T2DM	**Monotherapy:** Met+SU **LDC:** Met+SU	2005–2006	Morisky‐Green questionnaire	Monotherapy (49% high compliance) LDC (50% high compliance)	BMI, glycemic control
Cheong, 2008	USA	Retrospective cohort study	200831 patients with T2DM	**LDC:** glyburide+ Met, rosiglitazone+ Met, glipizide+ Met **FDC:** glyburide+ Met, rosiglitazone+ Met, glipizide+ Met	2000–2004	MPR	LDC (78.2%), FDC (78.6%)	Drug costs
Vanderpoel, 2004	USA	Retrospective cohort study	16,928 patients with T2DM	**Monotherapy:** rosiglitazone, Met **FDC:** rosiglitazone+ Met **LDC:** rosiglitazone+ Met	May 1, 2002, to February 29, 2004	MPR	**Monotherapy Cohorts**:	Patient age, pill burden, drug costs
							**Monotherapy/Monotherapy**: ○ Preindex MPR: 90% ○ Postindex MPR: 89% **Monotherapy/LDC**: ○ Preindex MPR: 80% ○ Postindex MPR: 70% **Monotherapy/FDCT**: ○ Preindex MPR: 87% ○ Postindex MPR: 83% **Dual Therapy Cohorts**:	
							**Dual/Dual**: ○ Preindex MPR: 84% ○ Postindex MPR: 83% **Dual/FDCT**: ○ Preindex MPR: 79% ○ Postindex MPR: 82%	

Abbreviations: DPP‐4i, dipeptidyl peptidase‐4 inhibitor; FDC, fixed dose combination; GM‐IR, glimepiride/metformin immediate release; GM‐SR, glimepiride/metformin sustained release; LDC, loose dose combination; MEMS, medication event monitoring system; Met, metformin; MPR, medication possession ratio; OHA, oral antihyperglycemic agent; PDC, proportion of days covered; SGLT‐2i, sodium‐glucose co‐transporter‐2 inhibitors; SU, sulfonylurea; T2DM, Type 2 Diabetes; TZD, thiazolidinediones; UK, United Kingdom; UK, United Kingdom; USA, United States of America.

### 4.3 Combination Antihyperglycemic Drugs

Included studies utilized a range of approaches to evaluate patients' adherence to combined diabetes medications. The evaluation of therapeutic options for T2DM involved comparing either individual drug treatment versus combinations or different types of combination therapies. Various forms of combination therapy utilizing oral antihyperglycemic agents (OHAs), including fixed‐dose combination (FDC) and loose‐dose combination (LDC), were utilized. Fixed‐dose antihyperglycemic drugs represent a cornerstone in the pharmacological management of diabetes mellitus, aiming to mitigate the deleterious effects of hyperglycemia. Comprising combinations of different classes of medications in predetermined dosages, these formulations offer a multifaceted approach to glycemic control by targeting distinct pathophysiological pathways implicated in diabetes. Such combinations, often including biguanides, sulfonylureas (SU), dipeptidyl peptidase‐4 (DPP‐4) inhibitors, sodium‐glucose cotransporter‐2 (SGLT‐2) inhibitors, thiazolidinediones (TZDs), and glucagon‐like peptide‐1 (GLP‐1) receptor agonists, not only demonstrate synergistic effects but also streamline medication regimens, potentially enhancing patient adherence and therapeutic outcomes. Consequently, the judicious selection and utilization of fixed‐dose antihyperglycemic agents stand as pivotal strategies in the clinical armamentarium against diabetes, demanding consideration of individual patient profiles and therapeutic goals [31]. A loose‐dose combination antihyperglycemic drug combines multiple diabetes medications in flexible doses, allowing for individualized adjustments based on patient needs. Unlike fixed‐dose combinations, where doses are predetermined, this approach offers flexibility in tailoring the dosage of each component to achieve optimal glycemic control while minimizing side effects. It typically includes various classes of antihyperglycemic agents like biguanides, sulfonylureas, DPP‐4 inhibitors, SGLT‐2 inhibitors, thiazolidinediones, and GLP‐1 receptor agonists, enabling personalized treatment strategies tailored to each patient's unique requirements [32].

#### 4.3.1 Adherence to Dual (Loose‐Dose) Antihyperglycemic Drugs

A cross‐sectional study conducted by Satirapoj et al., adherence to medication regimens among 659 T2DM patients came under scrutiny. The study specifically focused on comparing adherence patterns between SU monotherapy and SU in combination with Met therapy. Compliance assessments were conducted through a structured Self‐Report Adherence and Barriers Questionnaire, offering insights into patient‐reported adherence behaviors. Notably, the study reported an overall compliance rate of 52.2%, with no significant variance observed between the SU monotherapy and combination therapy groups. However, factors such as hypoglycemia and medication side effects emerged as pivotal influencers on adherence dynamics. Through a retrospective cohort study encompassing a vast cohort of 33,849 T2DM patients, Gordon et al. Gordon et al. [22] explored adherence trends across diverse OHA regimens. The study evaluated adherence to monotherapy, dual therapy, and triple therapy regimens, employing the robust MPR as a metric for adherence assessment. The findings showcased varied adherence rates across different therapy modalities, with monotherapy exhibiting an adherence rate of 81.6%, dual therapy at 80.8%, and triple therapy also at 80.8%. Noteworthy factors influencing adherence encompassed hypoglycemia rates, patient age, and baseline HbA1c levels, signifying the multifaceted nature of adherence determinants. A cross‐sectional study conducted among 294 T2DM patients in Spain, Yurgin et al. Yurgin et al. [30] embarked on a meticulous journey to explore adherence behaviors concerning monotherapy and dual therapy regimens, particularly focusing on the combination of Met and SU. The study employed the Morisky‐Green questionnaire, a validated tool for assessing medication adherence, to delve into adherence nuances. Encouragingly, the study reported commendable compliance rates, with monotherapy and LDC therapy demonstrating high adherence rates of 49% and 50%, respectively. Based on their study, there was almost no significant difference between adherence in monotherapy and LDC. Noteworthy influencers on adherence included variables such as BMI and glycemic control, underlining the intricate interplay of clinical and demographic factors in shaping adherence behaviors.

#### 4.3.2 Comparison of Adherence to Loose Dose and Fixed‐Dose Antihyperglycemic Drugs

FDC therapy has garnered significant attention in the management of T2DM, offering simplified medication regimens and potentially enhancing patient adherence. A retrospective cohort study conducted by Böhm et al, [18] in Germany assessed 990 patients with T2DM from 2013 to 2017, comparing FDC therapy with a combination of sitagliptin and metformin (Met) against LDC therapy. The study revealed increased adherence in the FDC group over 3 years, with adherence rates rising from 22% in year 1 to 25% in year 2 and further to 29% in year 3. Factors such as pill burden, concomitant diseases, and baseline adherence were identified as influencers of adherence. Similarly, Rombopoulos et al, [26], in Greece, investigated the adherence to Vildagliptin/Met FDC therapy compared to a LDC therapy. The study, spanning 24 weeks, demonstrated significantly higher adherence rates in the FDC group (98.9%) compared to the LDC group (84.6%). Factors such as difficulty remembering medication names and swallowing issues were highlighted as factors affecting adherence. Moreover, a study by Colombo [20] in Italy explored adherence across various monotherapy and combination therapy regimens. It was identified that Met+pioglitazone as having the highest adherence among FDC therapies (75.5%), with cost‐effectiveness being a notable factor. Another study from study examined a wide array of monotherapy agents and compared FDC like Metformin + SU, Fenformin + SU, Metformin + Rosiglitazone, and Metformin + Pioglitazone against LDC such as Biguanides + SU, Biguanides + TZD, and SU + TZD during 2008. The study reported varying medication possession ratios (MPR) with monotherapy at 68.5%, FDC at 60.3%, and LDC at 43.9%, reflecting differences in adherence across treatment regimens [21]. Within an extensive retrospective cohort study encompassing a staggering cohort of 200,831 T2DM patients in the USA, Cheong et al. Cheong et al. [19] in an exploration of adherence patterns pertaining to dual therapy regimens. The study evaluated adherence to various dual therapy combinations, including glyburide + Met, rosiglitazone + Met, and glipizide + Met. Adherence rates were quantified using the MPR metric, offering insights into medication possession dynamics. Notably, the study revealed a commendable adherence rate of 78.2% for dual therapy regimens. A significant determinant influencing adherence patterns emerged in the form of drug costs, elucidating the pivotal role of economic factors in shaping medication adherence behaviors. Another study from USA involving 23,361 T2DM patients and focused on combinations like DPP‐4i + Biguanides, DPP‐4i + TZD, Meglitinides + Biguanides, SU + TZD, SU + Biguanides, and TZD + Biguanides between 2009 and 2013. The research found that the medication possession ratio (MPR) was 67.9% in FDC groups compared to 73.4% in LDC groups, highlighting varying adherence patterns across different medication combinations [24]. Other similar research from USA by Thayer (2010) [28] and Vanderpoel (2004) [29] separately studied T2DM patients. Thayer and colleagues [28] examined 16,490 patients treated with SU and Rosiglitazone, comparing a FDC (Rosiglitazone/Glimepiride) with a LDC (SU and Rosiglitazone). The study noted a slight improvement in MPR with the FDC. Vanderpoel's study [29] also investigated 16,928 patients using Rosiglitazone and Metformin as monotherapy from 2002 to 2004 and the pre and post‐index MPR were demonstrated to be improved during study period. A retrospective longitudinal cohort study from Japan involving 16,057 patients with T2DM explored the comparative effectiveness of FDC versus LDC. The study analyzed various combinations such as TZD + Biguanides, TZD + SU, TZD + DPP‐4i, DPP‐4i + Biguanides, and Alpha‐glucosidase + Glinide over the period from 2011 to 2015. The MPR was notably higher in FDC groups (ranging from 70.4% to 75.6%) compared to LDC groups (ranging from 55.7% to 66.2%), as reported by Japan Medical Data Center and Medical Data Vision [25].

#### 4.3.3 Factors Influencing Adherence of Patients to Combination Therapy

Numerous factors have been elucidated in academic literature regarding patient adherence to combination therapies in the management of T2DM. These factors, identified across multiple studies, play pivotal roles in influencing patients' ability to adhere effectively to prescribed medication regimens, thus impacting treatment outcomes and overall disease management [18, 19, 20, 21, 22, 23, 24, 25, 26, 27, 28, 29, 30]. Foremost among these factors is glycemic control, a cornerstone of diabetes management. Extensively explored in six distinct studies, glycemic control encompasses parameters such as hypoglycemia [21, 22, 24, 27, 28, 30] and HbA1C levels [22]. Patients' adherence to combination therapies hinges significantly on their ability to maintain optimal glycemic levels, as deviations can lead to adverse health consequences and undermine treatment efficacy. Another critical determinant of adherence is weight management [27] and BMI [22, 30]. This factor, underscored in three studies, underscores the intricate interplay between medication adherence and metabolic health. Patients grappling with weight issues or elevated BMI may encounter challenges in adhering to combination therapies, potentially compromising treatment outcomes and exacerbating disease progression. Economic considerations, particularly drug costs, emerge as a salient factor shaping patient adherence behaviors. Addressed in five studies, the affordability and accessibility of prescribed medications significantly influence patients' adherence patterns. Notably, findings suggest that the relative affordability of FDC therapies compared to dual antihyperglycemic combinations positively impacts adherence, highlighting the nuanced relationship between cost dynamics and treatment adherence [19, 20, 24, 25, 29]. Furthermore, the complexity of medication regimens poses a formidable barrier to adherence. The number of OHAs administered to patients inversely correlates with adherence levels, as evidenced by findings from three studies. Patients contending with multiple medications face heightened pill burden [18, 21, 25, 28, 29], increased dosing frequency [23], and cognitive challenges associated with medication recall [26], all of which impede adherence and compromise treatment efficacy. Demographic factors, including baseline adherence levels [18], age, and sex [21, 22, 25, 28, 29], also exert a discernible influence on patient adherence behaviors. Explored in two studies, these factors underscore the need for tailored interventions to address the unique adherence challenges faced by diverse patient populations. Moreover, physiological parameters such as changes in total cholesterol levels have been implicated in influencing adherence behaviors, as demonstrated by findings from one study [22].

## 5 Improvement of Medication Adherence Using Digital Health Technology and Artificial Intelligence

Emerging digital health solutions—including mobile applications, telemedicine platforms, and wearable devices—offer promising avenues to enhance medication adherence in patients with T2DM. These technologies can facilitate real‐time monitoring of adherence behaviors, deliver personalized medication reminders, and support continuous patient engagement [33]. In parallel, artificial intelligence (AI) tools have the potential to analyze large datasets to predict non‐adherence, identify at‐risk patients, and enable clinicians to implement tailored interventions [34]. Although our scoping review primarily synthesizes existing literature on adherence patterns to oral combination therapies, it is important to acknowledge that the integration of digital health technologies and AI represents a critical frontier for future research. Preliminary evidence suggests that these innovative approaches can address barriers such as regimen complexity and patient forgetfulness, ultimately improving clinical outcomes [35].

Digital health technologies, such as mobile applications, wearable devices, and telehealth platforms, have shown significant potential in improving medication adherence. Mobile apps can send medication reminders, track adherence, and provide educational content tailored to individual patient needs. For example, a study by demonstrated that mobile health (mHealth) interventions, including medication reminders and educational content, significantly improved adherence rates among patients with chronic conditions, including diabetes [36]. Similarly, wearable devices, such as smartwatches, can monitor physiological parameters like blood glucose levels and physical activity, providing feedback that encourages patients to adhere to their treatment plans [37]. Continuous glucose monitors (CGMs) like the FreeStyle Libre and Dexcom G6 track glucose levels by utilizing a small sensor placed just beneath the skin, analyzing interstitial fluid. These devices deliver real‐time glucose readings and trend analysis without requiring traditional fingerstick calibration. The accuracy of glucose measurement is reflected in the mean absolute relative difference (MARD), with the FreeStyle Libre at 9.2% and the Dexcom G6 at 9.0%. Their ease of use and elimination of frequent needle sticks contribute to high user satisfaction [38, 39, 40].

Research has demonstrated that telehealth interventions can lead to significant improvements in medication adherence and glycemic control among patients with T2DM. For instance, a systematic review and meta‐analysis of randomized controlled trials evaluated the impact of telehealth on blood glucose management in individuals with diabetes. The study found that telehealth interventions were associated with a significant reduction in HbA1c levels, indicating enhanced glycemic control. The methodology involved comprehensive searches of electronic databases to identify relevant studies, followed by data extraction and analysis to assess the effectiveness of telehealth interventions on HbA1c reduction [41]. Similarly, another systematic review and meta‐analysis focused on the impact of diabetes self‐management education and support (DSMES) applications on medication adherence in patients with T2DM. This analysis revealed that DSMES app interventions significantly improved medication adherence compared to usual care. The methodology included searching multiple databases for interventional studies, assessing study bias, and conducting meta‐analyses to determine the effect of DSMES apps on adherence and clinical outcomes [42].

AI is increasingly being integrated into diabetes management, offering promising avenues to enhance medication adherence and glycemic control in patients with T2DM. A recent randomized clinical trial investigated the effectiveness of a voice‐based conversational AI application in managing basal insulin therapy for adults with T2DM. The study found that participants using the AI application achieved optimal insulin dosing more quickly, exhibited higher insulin adherence, experienced better glycemic control, and reported reduced diabetes‐related emotional distress compared to those receiving standard care. These findings suggest that AI‐driven interventions can significantly enhance medication adherence and glycemic control in T2DM patients [43] (Table 3).

**Table 3:** Digital health technologies and AI for enhancing medication adherence in T2DM.

Technology	Functionality	Key findings	References
Mobile applications (mHealth)	Medication reminders, adherence tracking, patient education	Improved adherence rates in chronic conditions, including diabetes	Company‐Bezares, Aretio‐Pousa [36]
Wearable devices (e.g., smartwatches)	Monitors blood glucose, physical activity, and vital signs	Encourages adherence through real‐time feedback	Jafleh et al. [37]
Continuous glucose monitors (CGMs)	Tracks glucose levels in interstitial fluid, provides real‐time data	FreeStyle Libre (MARD: 9.2%), Dexcom G6 (MARD: 9.0%) enhance user satisfaction and adherence	Rodriguez‐León et al. [38, 39, 40]
Telehealth interventions	Remote patient monitoring, virtual consultations, digital education	Systematic reviews show reduced HbA1c levels, improved adherence rates	Getie et al. [41, 42]
Diabetes self‐management education & support (DSMES) apps	Provides digital education, self‐monitoring support	Meta‐analysis found significant adherence improvements over standard care	Nkhoma et al. [42]
AI‐based conversational Agents	Virtual AI‐driven assistance for medication and therapy management	RCT found AI‐assisted insulin dosing led to improved adherence, glycemic control, and reduced distress	Nayak et al. [43]
Predictive AI models	Identifies at‐risk patients, predicts non‐adherence, supports clinicians	AI models enhance personalization and early intervention strategies	Yismaw et al. [34]

## 6 Discussion

The scoping review conducted on patient adherence for oral combination therapies in diabetes management offers a comprehensive insight into the complexities surrounding this crucial aspect of healthcare. As the prevalence of diabetes continues to rise globally, effective management strategies become imperative in mitigating the burden imposed by this chronic condition. Combination therapies have emerged as pivotal tools in achieving optimal glycemic control and reducing associated complications. However, the success of these therapies is contingent upon patient adherence, which remains a significant challenge in clinical practice [44].

Unlike previous reviews that have often focused on isolated aspects or specific regions, our work uniquely encompasses a diverse range of therapeutic regimens and patient populations. This broad approach has enabled us to identify novel insights into the multifactorial determinants of adherence—including clinical, economic, and psychosocial factors—and to examine differences between fixed‐dose and loose‐dose combination therapies. By addressing these underexplored areas, our review not only updates the current understanding of adherence challenges but also lays a foundation for future research to further investigate emerging interventions.

The review underscores the critical importance of glycemic control in influencing patient adherence to combination therapies for T2DM management. Optimal glycemic levels are essential for the efficacy of combination therapies, which often involve multiple medications targeting different pathways to achieve glycemic control [45]. Studies show that deviations from optimal glycemic levels, such as hypoglycemia or elevated HbA1C levels, significantly impact adherence to combination therapy regimens. Therefore, interventions aimed at improving adherence should prioritize strategies to maintain stable glycemic control, potentially through personalized treatment plans that consider the synergistic effects of combination therapies [46]. In the management of chronic ailments like diabetes, it's common for initial singular drug treatments to evolve into FDC therapies over time. This shift typically occurs due to alterations in the patient's therapeutic response or the advancing nature of the condition. Therefore, FDC therapy might represent the ultimate decision in the doctor‐patient interaction, seeking to optimize treatment for a chronic and dynamic condition like diabetes. This approach could potentially enhance patient compliance, particularly if it results in improved glycemic control [17, 47]. Also it was demonstrated that primary obstacles to attaining optimal medication adherence encompass hypoglycemia associated with the treatment regimen [48]. Within this framework, guidelines indicate that therapies involving DPP‐4 inhibitors or SGLT‐2 inhibitors are linked with minimal hypoglycemia risk. Conversely, treatments based on SU are associated with a moderate risk of hypoglycemia [49, 50].

This review demonstrated he interplay between weight management, BMI, and adherence to combination therapies highlights the complexity of managing T2DM with multiple medications. Patients grappling with weight issues or elevated BMI may face challenges in adhering to combination therapies, potentially compromising treatment outcomes. One of the significant obstacles to achieving optimal medication adherence is the potential for treatment‐induced weight gain [51]. In the cohort receiving solely SU treatment, there was a heightened occurrence of weight gain, whereas no significant alteration in body weight was observed following the administration of a combination of MET and SU. Consequently, in individuals with T2DM whose condition remains unregulated with SU alone, the inclusion of biguanides, contingent upon the patient's overweight status and the severity of their symptoms, may represent a suitable alternative [27]. It was shown that patients receiving OHA treatments linked to weight reduction and reduced hypoglycemia incidence generally exhibit improved adherence and better control of HbA1c levels. Notably, this pattern was evident across various OHA treatment sequences, indicating that adherence could play a progressively crucial role in glycemic control as patients transition from OHA monotherapy to dual‐therapy and triple‐therapy combinations [22]. DPP‐4i or SGLT‐2i treatments are linked to weight loss or maintenance, contrasting with TZD regimens, which lead to weight gain. On the other hand, therapies based on SU are associated with weight gain [22]. Additionally, research has demonstrated an interconnection between BMI and glycemic control in diabetes management [52]. The most reliable predictors for not attaining optimal glycemic control are treatment compliance and BMI [53]. These factors, heavily influenced by patient behavior, underscore the pivotal role patients play in achieving target clinical outcomes. Therefore, clinicians must persist in educating patients about the significance of both weight management and treatment adherence, emphasizing their direct correlation with clinical outcomes, including HbA1c control [54].

The affordability and accessibility of combination therapies play a pivotal role in shaping patient adherence behaviors towards their treatment regimen [55]. When these therapies are financially and logistically within reach for patients, they are more likely to adhere to their prescribed regimen consistently. Conversely, barriers such as high costs or limited availability can hinder patients from obtaining or continuing their combination therapies as directed, leading to lapses in adherence. Therefore, ensuring the affordability and accessibility of these treatments is essential in fostering positive adherence behaviors and ultimately improving health outcomes for patients with chronic conditions. Studies suggest that the relative affordability of FDC therapies compared to dual antihyperglycemic combinations positively impacts adherence [19, 20, 56]. Therefore, efforts to improve adherence to combination therapies should include initiatives to reduce the financial burden of medication costs, such as insurance coverage or patient assistance programs. Additionally, healthcare providers should consider cost‐effective combination therapy options when prescribing medications [56].

The management of T2DM presents a significant challenge due to the complexity of medication regimens involved. Patients undergoing combination therapies often face hurdles in adhering to their treatment plans. The multifaceted nature of T2DM requires a diverse range of medications, each with varying dosing schedules, administration requirements, and potential side effects. Moreover, the progressive nature of the disease necessitates adjustments to the medication regimen over time, further complicating treatment adherence [57]. Factors such as forgetfulness, pill burden, medication costs, and fear of side effects contribute to non‐adherence rates. To address this challenge, healthcare providers must prioritize patient education, simplify medication regimens whenever possible, and foster open communication to address patient concerns and barriers to adherence effectively. By doing so, healthcare professionals can enhance treatment outcomes and improve the quality of life for individuals managing T2DM. Patients contending with multiple medications face challenges such as pill burden, increased dosing frequency, and cognitive difficulties associated with medication recall [18, 23, 26]. Simplifying medication regimens through the use of FDC therapies or employing strategies to improve medication adherence, such as medication reminders or pill organizers, may help overcome these barriers and improve adherence to combination therapy regimens [58].

Demographic factors, including baseline adherence levels and age, exert a discernible influence on patient adherence behaviors in the context of combination therapies [18, 22]. Tailored interventions that take into account the unique adherence challenges faced by diverse patient populations receiving combination therapies are essential for improving treatment outcomes. Healthcare providers should consider individual patient characteristics and preferences when designing adherence interventions, particularly in the context of combination therapy regimens [59].

Understanding the multifaceted nature of patient adherence is crucial for healthcare providers in optimizing diabetes management. Clinicians must recognize the barriers patients face in adhering to combination therapies and tailor interventions accordingly [60]. Simplifying medication regimens, addressing economic barriers through access to affordable treatment options [61], and providing education and support to enhance patient understanding and motivation [62] are essential strategies for improving adherence. Furthermore, incorporating patient preferences and individualized treatment plans can promote patient‐centered care approaches, fostering a collaborative relationship between healthcare providers and patients [63]. A comprehensive meta‐analysis has revealed a noteworthy effect of SGLT‐2i on lipid profiles. Specifically, these inhibitors have been shown to markedly elevate high‐density lipoprotein (HDL) levels. Additionally, the analysis demonstrated a significant association between SGLT‐2 inhibitors and increased levels of low‐density lipoprotein (LDL) [64]. Research has substantiated the efficacy of GLP‐1 receptor agonists (GLP‐1 RAs) in diminishing plasma concentrations of total cholesterol, triglycerides, and low‐density lipoprotein cholesterol among diabetic individuals. Furthermore, sitagliptin demonstrated a capacity to lower intestinal cholesterol absorption in obese, insulin‐resistant mice. This suggests an additional potential mechanism that could contribute to the enhancement of cholesterol metabolism [64]. Of particular interest, the combined administration of GLP‐1 RAs and SGLT2i resulted in a noteworthy reduction in LDL cholesterol levels among diabetic patients, surpassing the effects observed with monotherapy [65].

In conclusion, patient adherence to oral combination therapies in diabetes management is a multifaceted issue influenced by various factors spanning clinical, economic, and psychosocial domains. Recognizing and addressing these factors are essential for optimizing treatment outcomes and reducing the burden of diabetes on individuals and healthcare systems. By adopting a patient‐centered approach and leveraging innovative strategies, healthcare providers can empower patients to adhere effectively to prescribed medication regimens, ultimately improving overall health outcomes and quality of life for individuals with diabetes.

## 7 Limitations and Strengths

This scoping review provides a comprehensive exploration of patient adherence to oral combination therapies in T2DM management. However, it is important to acknowledge both its limitations and strengths to offer a balanced perspective and guide future research. One limitation of this review is the variability in adherence measurement methods across the included studies. Adherence was assessed using tools such as MPR, PDC, self‐report questionnaires, and MEMS. This heterogeneity makes it challenging to directly compare adherence rates across studies and may introduce inconsistencies in the interpretation of results. Additionally, the inclusion of studies from diverse geographical regions—such as Germany, Thailand, South Korea, the UK, Greece, Italy, Spain, Japan, and the USA—introduces variability in healthcare systems, cultural attitudes, and socioeconomic factors, which may limit the generalizability of the findings. Furthermore, while the review identified several clinical and economic factors influencing adherence, there was limited exploration of psychosocial factors such as patient beliefs, attitudes, and social support systems, which are known to play a significant role in medication adherence.

Despite these limitations, the review has several strengths. Its comprehensive scope provides a broad overview of patient adherence to oral combination therapies in T2DM management, encompassing a wide range of therapeutic regimens, adherence measurement methods, and geographical contexts. The review adhered to the PRISMA‐ScR guidelines, ensuring methodological rigor and transparency. It also successfully identified critical factors influencing adherence, such as glycemic control, weight management, economic considerations, and regimen complexity, which can inform the development of targeted interventions. A unique strength of this review is its comparative analysis of FDC and LDC therapies, highlighting the potential advantages of FDCs in simplifying medication regimens and enhancing adherence.

## 8 Future Research Directions

While the scoping review provides valuable insights into patient adherence in diabetes management, several areas warrant further investigation. Longitudinal studies assessing adherence patterns over time, interventions aimed at improving adherence, and the impact of emerging therapies on patient adherence are avenues for future research. Additionally, exploring the role of digital health technologies, such as mobile applications and telemedicine, in enhancing adherence and facilitating remote monitoring and support for patients with diabetes could offer innovative solutions to address adherence challenges [66].

The review encompassed studies conducted in several countries, including Germany, Thailand, South Korea, the UK, Greece, Italy, Spain, Japan, and the USA. This broad geographical representation allows for a more comprehensive exploration of adherence patterns across different healthcare systems, cultural contexts, and patient populations. By including studies from diverse countries, the review captures the variability in healthcare practices, socioeconomic factors, and access to healthcare services, which can influence patient adherence behaviors. Comparing adherence rates and factors influencing adherence across different countries provides valuable insights into the global landscape of diabetes management and highlights the need for tailored interventions to address specific challenges faced by patients in different regions [67]. Additionally, the inclusion of studies from multiple countries underscores the universality of adherence issues in diabetes management and emphasizes the importance of developing strategies that are adaptable and culturally sensitive to promote optimal treatment outcomes worldwide [55].

Due to the diversity in measurement techniques utilized across the included studies, reaching a precise conclusion regarding patient adherence to combination therapies in diabetes management becomes challenging. Each study employed varied methods such as MPR, PDC, self‐report questionnaires, and MEMS, among others. This diversity introduces inconsistencies and potential biases in adherence assessment, making it difficult to compare adherence rates and draw definitive conclusions across studies. Therefore, while the review provides valuable insights into adherence patterns and influencing factors, the variability in measurement techniques underscores the need for caution in interpreting the findings and highlights the complexity of assessing adherence in clinical research. Further standardization and validation of adherence measurement methods are warranted to enhance the reliability and comparability of future studies in this area.

An important consideration in diabetes management is the role of prescriber permissions and barriers. The ability of cardiologists and other non‐endocrinologist healthcare providers to prescribe diabetes medications that afford cardiovascular benefits can significantly impact patient outcomes. According to recent research, there are notable prescriber pressures and barriers that can affect the prescription of these medications. These barriers include regulatory restrictions, lack of prescriber familiarity with diabetes medications, and institutional policies that limit prescriptive authority to certain specialists. These prescriber barriers can downstream affect patient adherence by limiting access to potentially beneficial medications and causing delays in initiating appropriate therapy. Addressing these barriers through policy changes, provider education, and collaborative care models is essential to ensuring that patients receive comprehensive and timely treatment. By enhancing prescriber flexibility and reducing bureaucratic obstacles, healthcare systems can improve adherence rates and overall patient outcomes [68].

It is essential to acknowledge the rapidly evolving landscape of diabetes management, which has seen the introduction of several new therapeutic options, including GLP‐1 receptor agonists (GLP1RA) and other novel agents. These therapies have demonstrated significant benefits in glycemic control, weight management, and cardiovascular outcomes, positioning them as crucial components of modern diabetes treatment protocols [69, 70]. GLP‐1RA therapies, in particular, improve glycemic control, promote weight loss, and have a low risk of hypoglycemia, except when used in combination with insulin or sulfonylureas [71]. Research indicates that GLP‐1RA users tend to be more adherent to their medication regimens compared to non‐users, with adherence rates of 75.2% and 71.5%, respectively [72]. Furthermore, a significant reduction in hospitalizations has been observed among adherent patients using GLP1‐RA therapies. These findings highlight the potential advantages of GLP‐1RAs in enhancing adherence and reducing adverse health outcomes, underscoring their role in contemporary diabetes management [73].

## Author Contributions

**Maryam Zarrinkamar:** conceptualization, investigation, methodology, visualization, writing – original draft, data curation. **Mojgan Geran:** conceptualization, investigation, writing – original draft, writing – review and editing, visualization, methodology, project administration, supervision, data curation. **Mohammad Geran:** data curation, methodology, visualization, conceptualization, investigation, writing – original draft.

## Conflicts of Interest

The authors declare no conflicts of interest.

## Transparency Statement

The lead author Mojgan Geran affirms that this manuscript is an honest, accurate, and transparent account of the study being reported; that no important aspects of the study have been omitted; and that any discrepancies from the study as planned (and, if relevant, registered) have been explained.

## Data Availability

The authors have nothing to report.

## References

[1] S. Mousavi , M. A. Khazeei Tabari , A. Bagheri , N. Samieefar , N. Shaterian , and R. Kelishadi , “The Role of p66Shc in Diabetes: A Comprehensive Review From Bench to Bedside,” Journal of Diabetes Research 2022 (2022): 1–15. 10.1155/2022/7703520 PMC9715346 36465704

[2] J. C. N. Chan , L.‐L. Lim , N. J. Wareham , et al., “The Lancet Commission on Diabetes: Using Data to Transform Diabetes Care and Patient Lives,” Lancet 396 (2020b): 2019–2082. 33189186 10.1016/S0140-6736(20)32374-6

[3] E. C. Dobrică , M. L. Banciu , V. Kipkorir , et al., “Diabetes and Skin Cancers: Risk Factors, Molecular Mechanisms and Impact on Prognosis,” World Journal of Clinical Cases 10 (2022): 11214–11225. 36387789 10.12998/wjcc.v10.i31.11214 PMC9649529

[4] D. Liang , X. Cai , Q. Guan , Y. Ou , X. Zheng , and X. Lin , “Burden of Type 1 and Type 2 Diabetes and High Fasting Plasma Glucose in Europe, 1990‐2019: A Comprehensive Analysis From the Global Burden of Disease Study 2019,” Frontiers in Endocrinology 14 (2023): 1307432. 38152139 10.3389/fendo.2023.1307432 PMC10752242

[5] K. L. Ong , L. K. Stafford , S. A. Mclaughlin , et al., “Global, Regional, and National Burden of Diabetes From 1990 to 2021, With Projections of Prevalence to 2050: A Systematic Analysis for the Global Burden of Disease Study 2021,” Lancet 402 (2023): 203–234. 37356446 10.1016/S0140-6736(23)01301-6 PMC10364581

[6] F. Firman and K. Lestari , “Cost Components Illness Analysis of Type 2 Diabetes Mellitus by Class of Care,” Indonesian Journal of Global Health Research 5 (2023): 1001–1012.

[7] M. A. Khazeei Tabari , R. Mirjalili , H. Khoshhal , et al., “Nature Against Diabetic Retinopathy: A Review on Antiangiogenic, Antioxidant, and Anti‐Inflammatory Phytochemicals,” Evidence‐Based Complementary and Alternative Medicine 2022 (2022): 1–18. 10.1155/2022/4708527 PMC8926515 35310030

[8] S. E. Inzucchi , R. M. Bergenstal , J. B. Buse , et al., “Management of Hyperglycemia in Type 2 Diabetes: A Patient‐Centered Approach: Position Statement of the American Diabetes Association (ADA) and the European Association for the Study of Diabetes (EASD),” Diabetes Care 35 (2012): 1364–1379. 22517736 10.2337/dc12-0413 PMC3357214

[9] F. Sugandh , M. Chandio , F. Raveena , et al., “Advances in the Management of Diabetes Mellitus: A Focus on Personalized Medicine,” Cureus 15 (2023): e43697. 37724233 10.7759/cureus.43697 PMC10505357

[10] N. Pourhabibi , B. Mohebbi , R. Sadeghi , et al., “Factors Associated With Treatment Adherence to Treatment Among in Patients With Type 2 Diabetes in Iran: A Cross‐Sectional Study,” Frontiers in Public Health 10 (2022): 976888. 36407991 10.3389/fpubh.2022.976888 PMC9667890

[11] E. Piragine , D. Petri , A. Martelli , V. Calderone , and E. Lucenteforte , “Adherence to Oral Antidiabetic Drugs in Patients With Type 2 Diabetes: Systematic Review and Meta‐Analysis,” Journal of Clinical Medicine 12 (2023): 1981. 36902770 10.3390/jcm12051981 PMC10004070

[12] M. J. Davies , D. A. D'Alessio , J. Fradkin , et al., “Management of Hyperglycemia in Type 2 Diabetes, 2018. A Consensus Report by the American Diabetes Association (ADA) and the European Association for the Study of Diabetes (EASD),” Diabetes Care 41 (2018): 2669–2701. 30291106 10.2337/dci18-0033 PMC6245208

[13] E. M. Vaughan , C. A. Johnston , D. J. Hyman , D. C. Hernandez , V. Hemmige , and J. P. Foreyt , “Dual Therapy Appears Superior to Monotherapy for Low‐Income Individuals With Newly Diagnosed Type 2 Diabetes,” Journal of Primary Care & Community Health 8 (2017): 305–311. 10.1177/2150131917745760 PMC5748290 29216790

[14] R. Schlosser , “Fixed‐Dose and Fixed‐Ratio Combination Therapies in Type 2 Diabetes,” Canadian Journal of Diabetes 43 (2019a): 440–444. 31375179 10.1016/j.jcjd.2019.05.005

[15] A. C. Tricco , E. Lillie , W. Zarin , et al., “PRISMA Extension for Scoping Reviews (PRISMA‐ScR): Checklist and Explanation,” Annals of Internal Medicine 169 (2018): 467–473. 30178033 10.7326/M18-0850

[16] S. Padhi , A. K. Nayak , and A. Behera , “Type II Diabetes Mellitus: A Review on Recent Drug Based Therapeutics,” Biomedicine & Pharmacotherapy = Biomedecine & Pharmacotherapie 131 (2020): 110708. 32927252 10.1016/j.biopha.2020.110708

[17] A. Kumar, 2021. Trace Amine‐Associated Receptor 1 Activation Regulates Glucose‐Dependent Insulin Secretion in Pancreatic Beta Cells In Vitro.

[18] A. K. Böhm , U. Schneider , J. Aberle , and T. Stargardt , “Regimen Simplification and Medication Adherence: Fixed‐Dose Versus Loose‐Dose Combination Therapy for Type 2 Diabetes,” PLoS One 16 (2021): e0250993. 33945556 10.1371/journal.pone.0250993 PMC8096115

[19] C. Cheong , J. C. Barner , K. A. Lawson , and M. T. Johnsrud , “Patient Adherence and Reimbursement Amount for Antidiabetic Fixed‐Dose Combination Products Compared With Dual Therapy Among Texas Medicaid Recipients,” Clinical Therapeutics 30 (2008): 1893–1907. 19014846 10.1016/j.clinthera.2008.10.003

[20] G. Colombo , G. Antonio Vittorino Gaddi , R. Elisa Rossi , B. Danilo Benedetto , and R. Marisa De Rosa , “Antidiabetic Therapy in Real Practice: Indicators for Adherence and Treatment Cost,” Patient Preference and Adherence 6 (2012): 653–661. 23055698 10.2147/PPA.S33968 PMC3461605

[21] A. Vittorino Gaddi , D. Benedetto , F. Capello , et al., “Oral Antidiabetic Therapy in a Large Italian Sample: Drug Supply and Compliance for Different Therapeutic Regimens,” Public Health 128 (2014): 70–76. 23969148 10.1016/j.puhe.2013.05.009

[22] J. Gordon , P. Mcewan , I. Idris , M. Evans , and J. Puelles , “Treatment Choice, Medication Adherence and Glycemic Efficacy in People With Type 2 Diabetes: A UK Clinical Practice Database Study,” BMJ Open Diabetes Research & Care 6 (2018): e000512. 10.1136/bmjdrc-2018-000512 PMC5942418 29755756

[23] J. D. Kim , C. Y. Park , B. Y. Cha , et al., “Comparison of Adherence to Glimepiride/Metformin Sustained Release Once‐Daily Versus Glimepiride/Metformin Immediate Release BID Fixed‐Combination Therapy Using the Medication Event Monitoring System in Patients With Type 2 Diabetes,” Clinical Therapeutics 40 (2018): 752–761.e2. 29729957 10.1016/j.clinthera.2018.04.002

[24] T. Lokhandwala , N. Smith , C. Sternhufvud , E. Sörstadius , W. C. Lee , and J. Mukherjee , “A Retrospective Study of Persistence, Adherence, and Health Economic Outcomes of Fixed‐Dose Combination vs Loose‐Dose Combination of Oral Anti‐Diabetes Drugs,” Journal of Medical Economics 19 (2016): 203–212. 26473990 10.3111/13696998.2015.1109518

[25] R. Nishimura , H. Kato , K. Kisanuki , et al., “Comparison of Persistence and Adherence Between Fixed‐Dose Combinations and Two‐Pill Combinations in Japanese Patients With Type 2 Diabetes,” Current Medical Research and Opinion 35 (2019): 869–878. 30460858 10.1080/03007995.2018.1551192

[26] G. Rombopoulos , M. Hatzikou , A. Athanasiadis , and M. Elisaf , “Treatment Compliance With Fixed‐Dose Combination of Vildagliptin/Metformin in Patients With Type 2 Diabetes Mellitus Inadequately Controlled With Metformin Monotherapy: A 24‐Week Observational Study,” International Journal of Endocrinology 2015 (2015): 1–5. 10.1155/2015/251485 PMC4452314 26089879

[27] B. Satirapoj , T. Pratipanawatr , B. Ongphiphadhanakul , S. Suwanwalaikorn , Y. Benjasuratwong , and W. Nitiyanant , “Real‐World Evaluation of Glycemic Control and Hypoglycemic Events Among Type 2 Diabetes Mellitus Study (REEDS): A Multicentre, Cross‐Sectional Study in Thailand,” BMJ Open 10 (2020): e031612. 10.1136/bmjopen-2019-031612 PMC7045111 32051301

[28] S. Thayer , B. Arondekar , C. Harley , and T. E. Darkow , “Adherence to a Fixed‐Dose Combination of Rosiglitazone/Glimepiride in Subjects Switching From Monotherapy or Dual Therapy With a Thiazolidinedione and/Or a Sulfonylurea,” Annals of Pharmacotherapy 44 (2010): 791–799. 20371759 10.1345/aph.1M426

[29] D. R. Vanderpoel , M. A. Hussein , T. Watson‐Heidari , and A. Perry , “Adherence to a Fixed‐Dose Combination of Rosiglitazone Maleate/Metformin Hydrochloride in Subjects With Type 2 Diabetes Mellitus: A Retrospective Database Analysis,” Clinical Therapeutics 26 (2004): 2066–2075. 15823770 10.1016/j.clinthera.2004.12.018

[30] N. R. Yurgin , K. S. Boye , T. Dilla , N. L. Suriñach , and X. B. Llach , “Physician and Patient Management of Type 2 Diabetes and Factors Related to Glycemic Control in Spain,” Patient Preference and Adherence 2 (2008): 87–95. 19920948 PMC2770414

[31] R. Schlosser , “Fixed‐Dose and Fixed‐Ratio Combination Therapies in Type 2 Diabetes,” Canadian Journal of Diabetes 43 (2019): 440–444. 31375179 10.1016/j.jcjd.2019.05.005

[32] K. Aoki , M. Nagakura , M. Taguri , et al., “Effect of Switching From an Anti‐Diabetic Loose Dose Combination to a Fixed Dose Combination Regimen at Equivalent Dosage for 6 Months on Glycemic Control in Japanese Patients With Type 2 Diabetes: A Pilot Study,” Journal of Clinical Medicine Research 9 (2017): 719–724. 28725321 10.14740/jocmr3067w PMC5505309

[33] D. Avoke , A. Elshafeey , R. Weinstein , C. H. Kim , and S. S. Martin , “Digital Health in Diabetes and Cardiovascular Disease,” Endocrine Research 49 (2024): 124–136. 38605594 10.1080/07435800.2024.2341146 PMC11484505

[34] M. B. Yismaw , C. Tafere , B. B. Tefera , et al., “Artificial Intelligence Based Predictive Tools for Identifying Type 2 Diabetes Patients at High Risk of Treatment Non‐Adherence: A Systematic Review,” International Journal of Medical Informatics 198 (2025): 105858. 40043515 10.1016/j.ijmedinf.2025.105858

[35] T. Fazila and N. Ahmad, Predictive Analytics and Digital Health Solutions: Enhancing Patient Adherence in Chronic Disease Management.

[36] F. Company‐Bezares and A. Aretio‐Pousa , “Mhealth Strategies to Improve Pharmacologic Adherence in Type 2 Diabetes Mellitus Patients: Systematic Review,” Farmacia hospitalaria: organo oficial de expresion cientifica de la Sociedad Espanola de Farmacia Hospitalaria 46 (2022): 59–68. 36520561

[37] E. A. Jafleh , F. A. Alnaqbi , H. A. Almaeeni , S. Faqeeh , M. A. Alzaabi , and K. Al Zaman , “The Role of Wearable Devices in Chronic Disease Monitoring and Patient Care: A Comprehensive Review,” Cureus 16 (2024): e68921. 39381470 10.7759/cureus.68921 PMC11461032

[38] S. Alva , T. Bailey , R. Brazg , et al., “Accuracy of a 14‐Day Factory‐Calibrated Continuous Glucose Monitoring System With Advanced Algorithm in Pediatric and Adult Population With Diabetes,” Journal of Diabetes Science and Technology 16 (2022): 70–77. 32954812 10.1177/1932296820958754 PMC8875061

[39] C. Rodriguez‐León , C. Villalonga , M. Munoz‐Torres , J. R. Ruiz , and O. Banos , “Mobile and Wearable Technology for the Monitoring of Diabetes‐Related Parameters: Systematic Review,” JMIR mHealth and uHealth 9 (2021): e25138. 34081010 10.2196/25138 PMC8212630

[40] N. Segev , L. N. Hornung , S. E. Tellez , et al., “Continuous Glucose Monitoring in the Intensive Care Unit Following Total Pancreatectomy With Islet Autotransplantation in Children: Establishing Accuracy of the Dexcom G6 Model,” Journal of Clinical Medicine 10, no. 9 (2021): 1893. 33925523 10.3390/jcm10091893 PMC8123839

[41] A. Getie , B. T. Amlak , T. Ayenew , and M. Gedfew , “Assessing the Impact of Telehealth on Blood Glucose Management Among Patients With Diabetes: A Systematic Review and Meta‐Analysis of Randomized Controlled Trials,” BMC Health Services Research 25 (2025): 285. 39979923 10.1186/s12913-025-12401-9 PMC11840977

[42] D. E. Nkhoma , C. J. Soko , K. J. Banda , D. Greenfield , Y.‐C. J. Li , and U. Iqbal , “Impact of Dsmes App Interventions on Medication Adherence in Type 2 Diabetes Mellitus: Systematic Review and Meta‐Analysis,” BMJ Health & Care Informatics 28 (2021): e100291. 10.1136/bmjhci-2020-100291 PMC8054079 33853862

[43] A. Nayak , S. Vakili , K. Nayak , et al., “Use of Voice‐Based Conversational Artificial Intelligence for Basal Insulin Prescription Management Among Patients With Type 2 Diabetes: A Randomized Clinical Trial,” JAMA Network Open 6 (2023): e2340232. 38039007 10.1001/jamanetworkopen.2023.40232 PMC10692866

[44] X. Ni , L. Zhang , X. Feng , and L. Tang , “New Hypoglycemic Drugs: Combination Drugs and Targets Discovery,” Frontiers in Pharmacology 13 (2022): 877797. 35865956 10.3389/fphar.2022.877797 PMC9295075

[45] D. Matthews , S. Del Prato , V. Mohan , et al., “Insights From VERIFY: Early Combination Therapy Provides Better Glycaemic Durability Than a Stepwise Approach in Newly Diagnosed Type 2 Diabetes,” Diabetes Therapy 11 (2020): 2465–2476. 32975711 10.1007/s13300-020-00926-7 PMC7547931

[46] J. L. S. Williams , R. J. Walker , B. L. Smalls , J. A. Campbell , and L. E. Egede , “Effective Interventions to Improve Medication Adherence in Type 2 Diabetes: A Systematic Review,” Diabetes Management 4 (2014): 29–48. 10.2217/dmt.13.62 PMC4157681 25214893

[47] M. Benford , G. Milligan , J. Pike , P. Anderson , J. Piercy , and S. Fermer , “Fixed‐Dose Combination Antidiabetic Therapy: Real‐World Factors Associated With Prescribing Choices and Relationship With Patient Satisfaction and Compliance,” Advances in Therapy 29 (2012): 26–40. 22246944 10.1007/s12325-011-0096-z

[48] A. T. Larkin , C. Hoffman , A. Stevens , A. Douglas , and Z. Bloomgarden , “Determinants of Adherence to Diabetes Treatment,” Journal of Diabetes 7 (2015): 864–871. 25565088 10.1111/1753-0407.12264

[49] S. E. Inzucchi , R. M. Bergenstal , J. B. Buse , et al., “Management of Hyperglycemia in Type 2 Diabetes, 2015: A Patient‐Centered Approach: Update to a Position Statement of the American Diabetes Association and the European Association for the Study of Diabetes,” Diabetes Care 38 (2015): 140–149. 25538310 10.2337/dc14-2441

[50] J. Kelwade , H. Parekh , V. Dukle , and B. Sethi , “How Many Oral Antidiabetic Drugs Before Insulin?,” Indian Journal of Endocrinology and Metabolism 21 (2017): 249–250. 28217528 10.4103/2230-8210.195994 PMC5240072

[51] K. S. Boye , S. Shinde , T. Kennedy‐Martin , S. Robinson , and V. T. Thieu , “Weight Change and the Association With Adherence and Persistence to Diabetes Therapy: A Narrative Review,” Patient Preference and Adherence 16 (2022): 23–39. 35023906 10.2147/PPA.S328583 PMC8747793

[52] K. Secnik , “The Association Between Body Mass Index and Glycemic Control in Patients With Type 2 Diabetes Across Eight Countries: A Literature Review,” Current Research in Diabetes & Obesity Journal 15 (2021), 10.19080/CRDOJ.2021.15.555904.

[53] M. K. Moon , K. Y. Hur , S. H. Ko , et al., “Combination Therapy of Oral Hypoglycemic Agents in Patients With Type 2 Diabetes Mellitus,” Korean Journal of Internal Medicine 32 (2017): 974–983. 29096431 10.3904/kjim.2017.354 PMC5668409

[54] M. J. Davies , V. R. Aroda , B. S. Collins , et al., “Management of Hyperglycaemia in Type 2 Diabetes, 2022. A Consensus Report by the American Diabetes Association (ADA) and the European Association for the Study of Diabetes (EASD),” Diabetologia 65 (2022): 1925–1966. 36151309 10.1007/s00125-022-05787-2 PMC9510507

[55] J. C. Miranda , S. A. Raza , B. Kolawole , et al., “Enhancing Diabetes Care in LMICs: Insights From a Multinational Consensus,” Pakistan Journal of Medical Sciences 39 (2023): 1899–1906. 37936776 10.12669/pjms.39.7.8881 PMC10626083

[56] A. K. Böhm , U. Schneider , and T. Stargardt , “Economic Effects of Fixed‐Dose Versus Loose‐Dose Combination Therapy for Type 2 Diabetes Patients,” Applied Health Economics and Health Policy 21 (2023): 109–118. 36310297 10.1007/s40258-022-00760-x PMC9834204

[57] A. A. Ayele , H. G. Tegegn , T. A. Ayele , and M. B. Ayalew , “Medication Regimen Complexity and Its Impact on Medication Adherence and Glycemic Control Among Patients With Type 2 Diabetes Mellitus in an Ethiopian General Hospital,” BMJ Open Diabetes Research & Care 7 (2019): e000685. 10.1136/bmjdrc-2019-000685 PMC6606061 31321061

[58] M. H. Elnaem , N. A. Irwan , U. Abubakar , S. A. Syed Sulaiman , M. E. Elrggal , and E. Cheema , “Impact of Medication Regimen Simplification on Medication Adherence and Clinical Outcomes in Patients With Long‐Term Medical Conditions,” Patient Preference and Adherence 14 (2020): 2135–2145. 33173282 10.2147/PPA.S268499 PMC7646472

[59] E. Menditto , V. Orlando , G. De Rosa , et al., “Patient Centric Pharmaceutical Drug Product Design‐The Impact on Medication Adherence,” Pharmaceutics 12 (2020): 44. 31947888 10.3390/pharmaceutics12010044 PMC7023035

[60] A. H. Y. Chan , V. Cooper , H. Lycett , and R. Horne , “Practical Barriers to Medication Adherence: What Do Current Self‐ or Observer‐Reported Instruments Assess?,” Frontiers in Pharmacology 11 (2020a): 572. 32477110 10.3389/fphar.2020.00572 PMC7237632

[61] N. Karki , K. Kandel , K. Shah , P. Prasad , and J. Khanal , “Combination Therapy in Diabetes Mellitus Patients Attending Outpatient Department in a Tertiary Care Centre: A Descriptive Cross‐Sectional Study,” Journal of Nepal Medical Association 60 (2022): 1016–1020. 10.31729/jnma.7642 PMC9795118 36705114

[62] G. Parati , S. Kjeldsen , A. Coca , W. C. Cushman , and J. Wang , “Adherence to Single‐Pill Versus Free‐Equivalent Combination Therapy in Hypertension: A Systematic Review and Meta‐Analysis,” Hypertension 77 (2021): 692–705. 33390044 10.1161/HYPERTENSIONAHA.120.15781

[63] L. D. Scherer , D. D. Matlock , L. A. Allen , et al., “Patient Roadmaps for Chronic Illness: Introducing a New Approach for Fostering Patient‐Centered Care,” MDM Policy & Practice 6 (2021): 23814683211019947. 34277949 10.1177/23814683211019947 PMC8255605

[64] S. Dar , A. K. Siddiqi , T. O. Alabduladhem , et al., “Effects of Novel Glucose‐Lowering Drugs on the Lipid Parameters: A Systematic Review and Meta‐Analysis,” Annals of Medicine & Surgery 77 (2022): 103633. 35637990 10.1016/j.amsu.2022.103633 PMC9142616

[65] C. Li , J. Luo , M. Jiang , and K. Wang , “The Efficacy and Safety of the Combination Therapy With GLP‐1 Receptor Agonists and SGLT‐2 Inhibitors in Type 2 Diabetes Mellitus: A Systematic Review and Meta‐Analysis,” Frontiers in Pharmacology 13 (2022): 838277. 35185588 10.3389/fphar.2022.838277 PMC8854770

[66] F. Aberer , D. A. Hochfellner , and J. K. Mader , “Application of Telemedicine in Diabetes Care: The Time Is Now,” Diabetes Therapy 12 (2021): 629–639. 33474646 10.1007/s13300-020-00996-7 PMC7816834

[67] C. M. Studer , M. Linder , and L. Pazzagli , “A Global Systematic Overview of Socioeconomic Factors Associated With Antidiabetic Medication Adherence in Individuals With Type 2 Diabetes,” Journal of Health, Population, and Nutrition 42 (2023): 122. 37936205 10.1186/s41043-023-00459-2 PMC10631092

[68] A. Sharma , H. Aziz , S. Verma , et al., “Permission to Prescribe: Do Cardiologists Need Permission to Prescribe Diabetes Medications That Afford Cardiovascular Benefit?,” Current Opinion in Cardiology 36 (2021): 672–681. 34173772 10.1097/HCO.0000000000000892

[69] F. Giorgino , A. Penfornis , V. Pechtner , R. Gentilella , and A. Corcos , “Adherence to Antihyperglycemic Medications and Glucagon‐Like Peptide 1‐Receptor Agonists in Type 2 Diabetes: Clinical Consequences and Strategies for Improvement,” Patient Preference and Adherence 12 (2018): 707–719. 29765207 10.2147/PPA.S151736 PMC5944456

[70] P. Gouda , S. Zheng , T. Peters , et al., “Clinical Phenotypes in Patients With Type 2 Diabetes Mellitus: Characteristics, Cardiovascular Outcomes and Treatment Strategies,” Current Heart Failure Reports 18 (2021): 253–263. 34427881 10.1007/s11897-021-00527-w

[71] N. B. Dalsgaard , A. Brønden , T. Vilsbøll , and F. K. Knop , “Cardiovascular Safety and Benefits of GLP‐1 Receptor Agonists,” Expert Opinion on Drug Safety 16 (2017): 351–363. 28102093 10.1080/14740338.2017.1281246

[72] A. Palanca , F. J. Ampudia‐Blasco , J. M. Calderón , et al., “Real‐World Evaluation of GLP‐1 Receptor Aganist Therapy Persistence, Adherence and Therapeutic Inertia Among Obese Adults With Type 2 Diabetes,” Diabetes Therapy 14 (2023): 723–736. 36847952 10.1007/s13300-023-01382-9 PMC10064368

[73] S. Ciardullo , L. Savaré , F. Rea , G. Perseghin , and G. Corrao , “Adherence to GLP1‐RA and SGLT2‐I Affects Clinical Outcomes and Costs in Patients With Type 2 Diabetes,” Diabetes/Metabolism Research and Reviews 40 (2024): e3791. 38549238 10.1002/dmrr.3791

